# A simple replacement method for a 7 Fr dedicated plastic stent in endoscopic ultrasound-guided hepaticogastrostomy

**DOI:** 10.1055/a-2081-9593

**Published:** 2023-05-26

**Authors:** Yuki Ishikawa-Kakiya, Hirotsugu Maruyama, Masafumi Yamamura, Kojiro Tanoue, Akira Higashimori, Shusei Fukunaga, Yasuhiro Fujiwara

**Affiliations:** Department of Gastroenterology, Osaka Metropolitan University Graduate School of Medicine, Osaka, Japan

Endoscopic ultrasonography-guided hepaticogastrostomy (EUS-HGS) is expected to become widespread in the future 1 . A 7 Fr dedicated plastic stent (Through and Pass, TYPE‐IT; Gadelius Medical Co. Ltd., Tokyo, Japan) ( Fig. 1 ) is often used to prevent serious adverse events, such as migration or obstruction of bile duct branches 2 3 . However, this stent has a pigtail structure on the stomach side, making it difficult to place a guidewire through the stent. Furthermore, inserting a guidewire into the side of the stent is associated with strong frictional resistance and may result in placement intraperitoneally. When removing the stent with grasping forceps, the tube may be left in the liver as it is readily fractured ( Fig. 2 ). This case demonstrates a method we devised for inserting a guidewire into a dedicated plastic stent for easy removal.

**Fig. 1:**
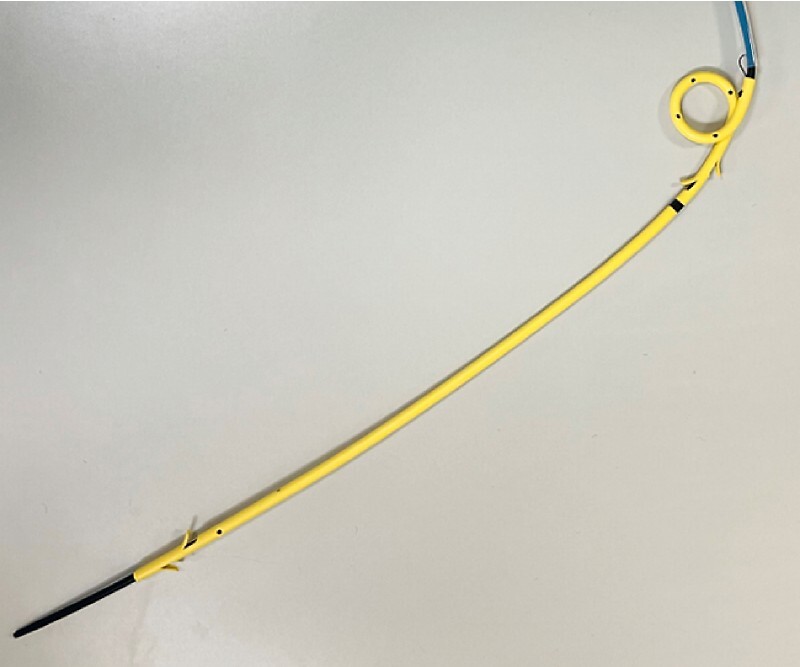
The 7 Fr dedicated plastic stent.

**Fig. 2:**
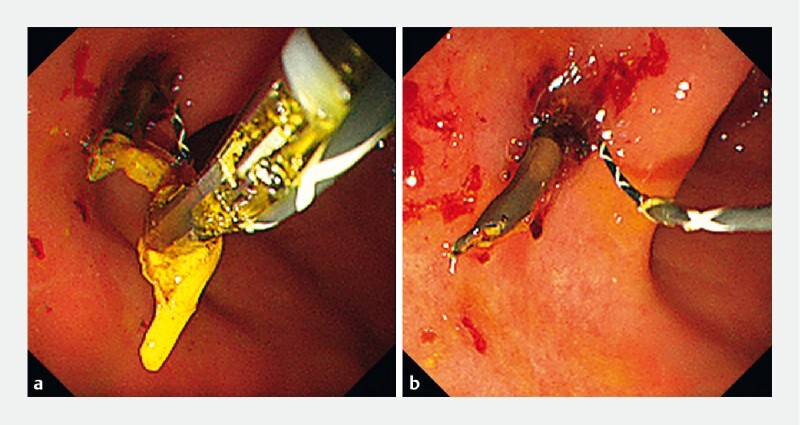
Endoscopic images of stent removal attempt. **a** The stent is grasped by forceps for removal. **b** The stent is left behind in the liver following fracture during the removal attempt.

A 75-year-old woman developed cholangitis from perihilar cholangiocarcinoma, which was not controlled with multiple transpapillary stents. EUS-HGS was performed, and after creation of the gastrobiliary fistula, the plastic stent was replaced with a metal stent.

For stent replacement, we inserted the guidewire into a 3.5 Fr catheter (PR-110Q-1; Olympus Medical Systems, Tokyo, Japan), which was grasped by a snare (SD-8P-1, Olympus) ( Fig. 3 ). We used a side-viewing duodenoscope (TJF 260; Olympus) and inserted the cannulated guidewire on the pigtail side ( Video 1 ). After guiding through the loop of the pigtail, the snare was opened, the distal end of the plastic stent was grasped ( Fig. 4 ), and the stent was pulled into the scope for straightening. Then, the guidewire was inserted into the bile duct and the stent was removed by pulling the snare. A cholangiogram was performed and the covered metal stent was placed.

**Fig. 3:**
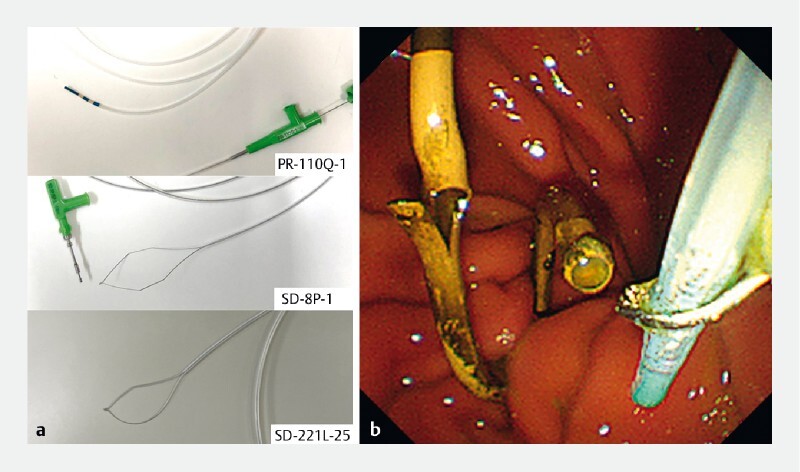
Method for stent removal. **a** The 3.5 Fr catheter (above) and snare (middle); a disposable electrosurgical snare (SD-221L-25; Olympus, Tokyo, Japan) can be used as a substitute (below). **b** The 3.5 Fr catheter grasped by the snare.

**Video 1** Easy replacement method for a 7 Fr dedicated plastic stent in endoscopic ultrasound-guided hepaticogastrostomy.


**Fig. 4:**
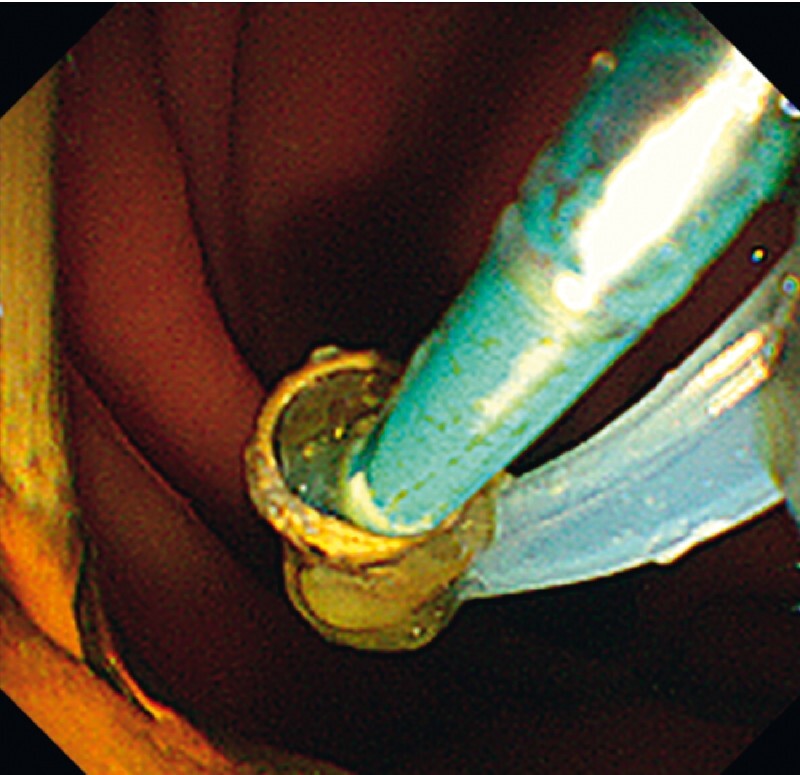
The guidewire was inserted into the loop of the pigtail, and the snare grasped the plastic distal end.

This method enables the guidewire to be reliably placed in the bile duct, enabling safe exchange of a dedicated plastic stent.

Endoscopy_UCTN_Code_TTT_1AS_2AD

## Notes

Endoscopy E-Videos

https://eref.thieme.de/e-videos
*E-Videos* is an open access online section of the journal *Endoscopy* , reporting on interesting cases and new techniques in gastroenterological endoscopy. All papers include a high-quality video and are published with a Creative Commons CC-BY license. Endoscopy E-Videos qualify for HINARI discounts and waivers and eligibility is automatically checked during the submission process. We grant 100% waivers to articles whose corresponding authors are based in Group A countries and 50% waivers to those who are based in Group B countries as classified by Research4Life (see: https://www.research4life.org/access/eligibility/ ). This section has its own submission website at https://mc.manuscriptcentral.com/e-videos
